# Knowledge, Beliefs and Practices Regarding Antiretroviral Medications for HIV Prevention: Results from a Survey of Healthcare Providers in New England

**DOI:** 10.1371/journal.pone.0132398

**Published:** 2015-07-06

**Authors:** Douglas S. Krakower, Catherine E. Oldenburg, Jennifer A. Mitty, Ira B. Wilson, Ann E. Kurth, Kevin M. Maloney, Donna Gallagher, Kenneth H. Mayer

**Affiliations:** 1 Division of Infectious Diseases, Beth Israel Deaconess Medical Center, Boston, MA, United States of America; 2 Harvard Medical School, Boston, Massachusetts, United States of America; 3 The Fenway Institute, Boston, Massachusetts, United States of America; 4 Harvard School of Public Health, Boston, Massachusetts, United States of America; 5 Brown University School of Public Health, Providence, Rhode Island, United States of America; 6 New York University, New York, New York, United States of America; 7 New England AIDS Education and Training Center, Boston, Massachusetts, United States of America; Centers for Disease Control and Prevention, UNITED STATES

## Abstract

**Background:**

Antiretroviral treatment for HIV-infection before immunologic decline (early ART) and pre-exposure chemoprophylaxis (PrEP) can prevent HIV transmission, but routine adoption of these practices by clinicians has been limited.

**Methods:**

Between September and December 2013, healthcare practitioners affiliated with a regional AIDS Education and Training Center in New England were invited to complete online surveys assessing knowledge, beliefs and practices regarding early ART and PrEP. Multivariable models were utilized to determine characteristics associated with prescribing intentions and practices.

**Results:**

Surveys were completed by 184 practitioners. Respondent median age was 44 years, 58% were female, and 82% were white. Among ART-prescribing clinicians (61% of the entire sample), 64% were aware that HIV treatment guidelines from the Department of Health and Human Services recommended early ART, and 69% indicated they would prescribe ART to all HIV-infected patients irrespective of immunologic status. However, 77% of ART-prescribing clinicians would defer ART for patients not ready to initiate treatment. Three-fourths of all respondents were aware of guidance from the U.S. Centers for Disease Control and Prevention recommending PrEP provision, 19% had prescribed PrEP, and 58% of clinicians who had not prescribed PrEP anticipated future prescribing. Practitioners expressed theoretical concerns and perceived practical barriers to prescribing early ART and PrEP. Clinicians with higher percentages of HIV-infected patients (aOR 1.16 per 10% increase in proportion of patients with HIV-infection, 95% CI 1.01–1.34) and infectious diseases specialists (versus primary care physicians; aOR 3.32, 95% CI 0.98–11.2) were more likely to report intentions to prescribe early ART. Higher percentage of HIV-infected patients was also associated with having prescribed PrEP (aOR 1.19, 95% CI 1.06–1.34), whereas female gender (aOR 0.26, 95% CI 0.10–0.71) was associated with having not prescribed PrEP.

**Conclusions:**

These findings suggest many clinicians have shifted towards routinely recommending early ART, but not PrEP, so interventions to facilitate PrEP provision are needed.

## Introduction

As there are 50,000 new infections in the U.S. annually [1], effective HIV prevention strategies are needed. Studies have demonstrated that antiretroviral medications can decrease HIV transmission when used as treatment by HIV-infected persons (“Treatment as Prevention”) or as pre-exposure chemoprophylaxis (“PrEP”) for HIV-uninfected persons at high risk for HIV acquisition. In 2011, the HIV Prevention Trials Network 052 Study (HPTN 052) demonstrated that early initiation of antiretroviral treatment (“early ART”) by the HIV-infected partner in serodiscordant couples reduced the risk of HIV transmission by 96% [2], and observational studies support the role of ART in decreasing HIV transmission on a community level [3]. As a result of HPTN 052 and studies suggesting that earlier initiation of ART confers personal health benefits [4, 5], HIV treatment guidelines from the Department of Health and Human Services (DHHS) were updated in 2012 to recommend ART for all HIV-infected persons irrespective of immunologic status if they were ready to initiate treatment [6]. Between 2011 and 2013, the US Centers for Disease Control and Prevention (CDC) released guidance for practitioners regarding PrEP prescribing based on studies demonstrating the efficacy of daily oral PrEP among at-risk populations [7–10], and in May 2014, formal CDC guidelines were published [11].

However, implementation of Treatment as Prevention and PrEP will depend on whether healthcare practitioners will prescribe early ART and PrEP to their patients. Prior studies suggested that HIV clinicians may not intend to prescribe early ART to all of their HIV-infected patients [12], and that few practitioners have prescribed PrEP [13]. One year prior to the publication of HPTN 052 and the recent update in treatment guidelines that endorse early ART, a minority (14%) of experienced HIV clinicians in Washington, D.C. and the Bronx, N.Y. indicated they would recommend ART for all patients irrespective of CD4+ count, even though most practitioners believed that early ART would decrease HIV transmission [12]. Some clinicians indicated concerns about potential toxicities from ART and development of drug resistant virus if ART is initiated too early. Studies conducted prior to the introduction of formal PrEP guidelines demonstrated that many healthcare practitioners reported positive attitudes towards PrEP, but that prescribing experience was uncommon and prescribing intentions were variable [13–20].

As studies have suggested that early ART and PrEP could decrease HIV incidence, but many practitioners have not adopted early ART and PrEP provision into practice, there is a need to ascertain whether clinician attitudes and practices regarding early ART have evolved since HPTN 052 and the updated HIV treatment guidelines, and to gauge recent prescribing experiences and intentions with PrEP. In New England, HIV care is provided in HIV specialty clinics and in primary care practices, so this region offers an opportunity to assess the beliefs and behaviors of HIV clinicians who practice in heterogeneous settings. New England also has a regional AIDS Education and Training Center, so some practitioners affiliated with this center could be expected to be early adopters of innovations such as early ART or PrEP, even prior to comprehensive guidelines from CDC. Therefore, the current study assessed knowledge, practices, and perceptions regarding early ART and PrEP among healthcare practitioners affiliated with the New England AIDS Education Training Center (NEAETC).

## Methods

### Recruitment and Sample Selection

Between September and December 2013, invitations to complete an anonymous online survey were emailed to healthcare practitioners who attended educational programs offered by the NEAETC. The NEAETC is one of 11 regional education centers funded by the U.S. Health Resources and Services Administration’s HIV/AIDS bureau [21]. Eligibility criteria for survey participants included NEAETC membership, age ≥ 18 years, and ability to read and understand English. Subjects were recruited from an NEAETC database containing practitioners’ email addresses, geographic location, gender, training background, years of experience providing care to HIV-infected patients, and monthly volume of HIV-infected patients. Invitations were emailed to all Medical Doctor (M.D.), Doctor of Osteopathy (D.O.), Physician Assistant (PA), or Advanced Nurse Practitioner (ANP) members with functional email addresses (n = 1637). Up to 4 reminder emails were sent to non-responders. Online surveys were administered using Qualtrics, a secure online survey platform. Participants were emailed $25.00 Amazon.com gift cards.

### Survey Development

Development of a 46-item survey was informed by a qualitative study assessing HIV clinicians’ perceived facilitators and barriers to implementing early ART and PrEP [15, 22]. Assessment items were adapted from prior quantitative studies of clinician practices regarding early ART [12] or PrEP [14] or were developed *de novo* using survey design methodology intended to maximize survey reliability [23]. The questionnaire was revised based on cognitive interviews with clinicians (n = 5) [24, 25] and feedback from external experts in HIV prevention (n = 2).

### Survey Domains and Measures

**Fig 1:**
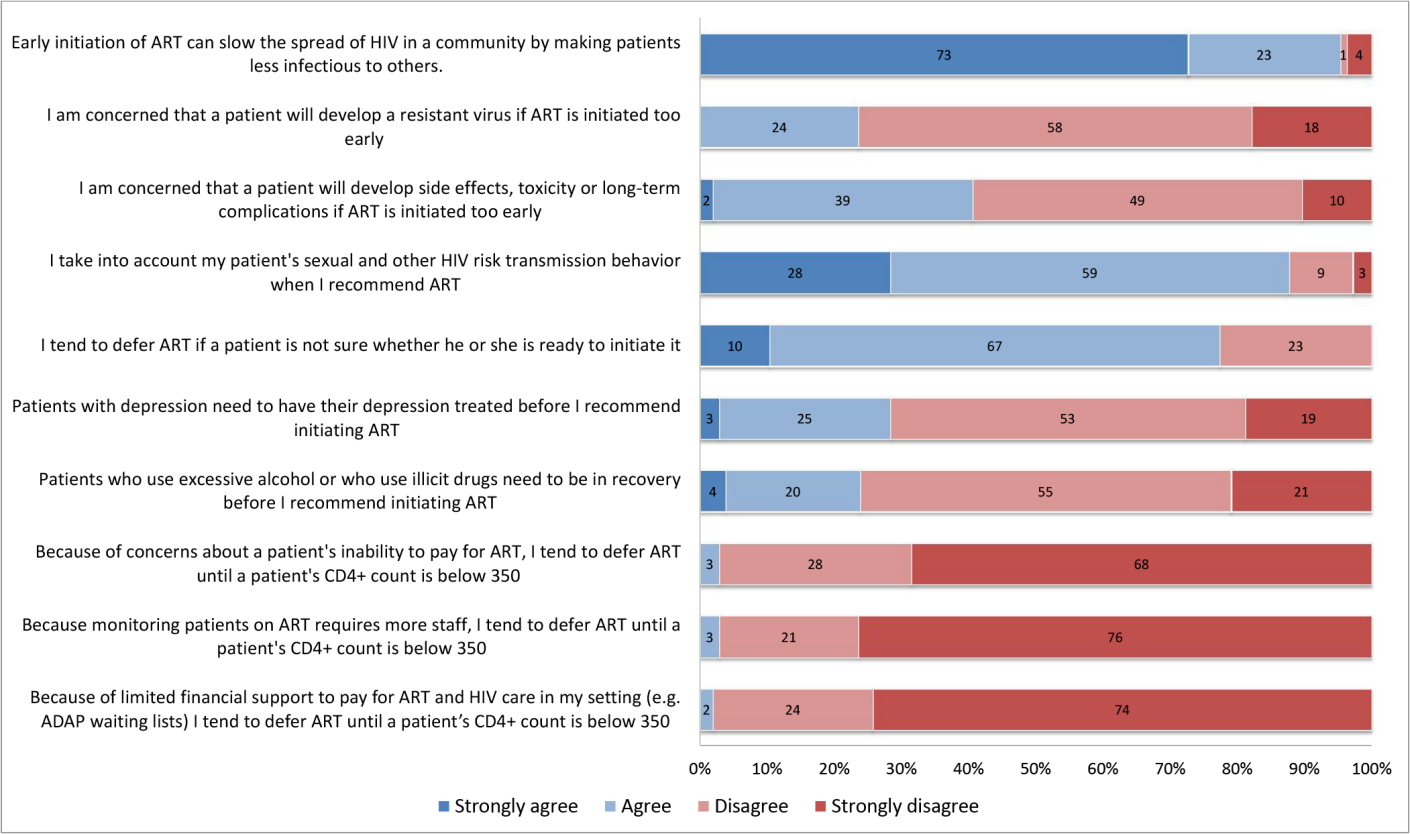
Clinician beliefs and reported practices regarding early antiretroviral treatment (n = 105), New England, 2013. Participants were presented with statements about early antiretroviral treatment and asked to indicate their degree of agreement (strongly agree, agree, disagree, strongly disagree). Numbers within bars represent the percentage of respondents selecting each response category. Blue shading represents agreement, whereas red shading represents disagreement. Data are restricted to clinicians who have prescribed antiretroviral therapy to at least 1 HIV-infected patient in the prior year.

Survey domains relating to early ART included knowledge of guideline recommendations, beliefs, prescribing intentions and practices, and experiences discussing early ART with patients. Knowledge was measured by asking participants to indicate whether or not DHHS guidelines recommended ART for all HIV-infected patients irrespective of CD4+ count (true, false, not sure). Beliefs were assessed by agreement (strongly agree, agree, disagree, strongly disagree) to 10 statements about the effectiveness and potential harms of early ART and patient and practice-level factors that could influence prescribing decisions [12] (Fig 1). Intentions to prescribe early ART to patients with various CD4+ counts and co-occurring medical conditions were measured through 10 brief patient scenarios [12]. For these 10 scenarios, participants were asked, “In which of the following scenarios would you generally recommend that ART be initiated for a typical HIV-infected patient?” Scenarios included patients with CD4+ count ≤200 cells/mm3, ≤350 cells/mm3, ≤500 cells/mm3, or all patients irrespective of CD4+ count, and patients with chronic hepatitis B, hepatitis C, HIV-associated nephropathy, HIV-associated dementia, pregnancy, or those receiving tuberculosis treatment, all irrespective of CD4+ count. Experiences with discussing early ART were assessed by asking clinicians to indicate, for patients with CD4+ count > 500 cells/μL, how frequently they recommended ART, whether patients objected to ART, and whether practitioners attempted to persuade objecting patients to initiate ART (always, often, sometimes, rarely, never, not applicable).

Domains relating to PrEP included awareness, knowledge of normative guidance, prescribing practices and intentions, beliefs, and perceived barriers to prescribing PrEP. Participants were presented with a definition of PrEP and asked if they had heard of PrEP (yes, no). Knowledge was assessed by statements about whether the Food and Drug Administration (FDA) had approved Truvada for use as PrEP and whether CDC had issued guidance for clinicians (true, false, not sure). Prescribing practices were measured by asking if any providers at a participant’s clinic/center are currently prescribing PrEP (yes, no, don’t know, refuse to answer, not applicable) and for how many patients the participant had ever prescribed PrEP. If participants had prescribed PrEP, they were asked to indicate for which groups of at-risk patients they had prescribed PrEP [14]. Prescribing intentions were measured by asking about future likelihood of prescribing PrEP (very unlikely, unlikely, likely, very likely). Beliefs were assessed by agreement to 8 statements about potential concerns regarding PrEP that were adapted from a prior survey conducted at Fenway Health (strongly agree, agree, neutral, disagree, strongly disagree) [14]. Clinicians were also asked to rate 7 potential barriers to prescribing PrEP (not a barrier, minor barrier, moderate barrier, major barrier).

### Data Analysis

A complete case analytic strategy was used for all analyses. Respondents were excluded from analyses of individual questions for which they did not provide responses. Participant demographics, practice characteristics, knowledge, beliefs, practices, and prescribing intentions regarding early ART and PrEP were described.

Multivariable logistic regression models for dichotomous outcomes and multivariable ordinal logistic regression models were fit to determine independent associations between practitioner-related covariates and outcomes of interest relating to early ART and PrEP. Outcomes for models included: (1) awareness of DHHS recommendations regarding early ART; (2) recommending initiation of ART for patients with CD4+ count > 500 cells/μL (on a Likert scale and modeled as an ordinal variable); (3) intentions to prescribe ART to all patients irrespective of CD4+ count; (4) awareness of CDC recommendations regarding PrEP; (5) having prescribed PrEP; and (6) likelihood of prescribing PrEP in the future (on a Likert scale and modeled as an ordinal variable), among clinicians who had not previously prescribed PrEP. The proportional odds assumption was verified for ordinal logistic regression models with a likelihood ratio test; both ordinal logistic regression models met the proportional odds assumption. Respondents from specialties that do not provide longitudinal care (e.g., emergency medicine) were excluded from analyses relating to PrEP prescribing, given the follow-up monitoring that is recommended with PrEP. Covariates in these models included clinician demographics and characteristics previously shown to be associated with antiretroviral prescribing patterns [26, 27]. To account for potential confounders, models were adjusted for all other covariates included in the models. Adjusted odds ratios (aOR) and 95% confidence intervals (CI) were calculated. Statistical analyses were conducted with Stata 13.0 (StataCorp, College Station, TX).

### Ethics Approval

All study procedures were approved by the Institutional Review Board at Beth Israel Deaconess Medical Center. Respondents indicated consent at the start of the online questionnaire after reviewing a written description of the risks and benefits of study participation.

## Results

### Participant Demographics and Practice Characteristics

Of 1637 invitations sent, 207 started and 184 completed the survey, for a response rate of 11% and a completion rate of 89%. Respondents’ median age was 44 years, 57% were female, and 82% were white (Table 1). Fourteen percent identified as gay, lesbian, or bisexual. Most clinicians practiced at public clinics (59%) in urban settings (78%). All 6 New England states were represented. A majority of respondents were nurse practitioners (24%), primary care physicians (22%), or infectious diseases specialists (21%). In terms of experience with caring for lesbian, gay, bisexual, or transgender persons, participants rated themselves as not experienced (2%), a little experienced (26%), moderately experienced (47%), or very experienced (22%). Clinicians had a median of 15% (interquartile range 5–45%) of patients in their panel with HIV-infection, and 11% of respondents reported that at least 90% of the patients in their care were infected with HIV. Participants had provided care to HIV-infected patients for a median of 10 years and reported a median of 17 HIV-infected patients under direct care. Of the entire sample, 112 practitioners were ART-prescribing clinicians, defined as having prescribed ART to at least 1 HIV-infected patient in the prior year.

**Table 1:** Demographics and practice characteristics of healthcare practitioners completing a survey regarding the use of antiretroviral medications for HIV prevention (n = 184), New England, 2013.

Clinician Characteristics		Median (IQR) or n (%)
Age, years		44 (35–55)
Female		103/181 (56.9)
Race	White	142/174 (81.6)
	Asian	21/174 (12.1)
	Black or African American	9/174 (5.2)
	Other	2/174 (1.2)
Hispanic or Latino/a origin		6/174 (3.4)
Gay, Lesbian, or Bisexual		26/178 (14.2)
Clinic type	Public	108/183 (59.0)
	Private	38/183 (20.8)
	Other	37/183 (20.2)
Employment Setting	Urban	141/182 (77.5)
	Suburban	31/182 (17.0)
	Rural	10/182 (5.5)
Location	Massachusetts	101/184 (54.9)
	Connecticut	35/184 (19.0)
	Rhode Island	22/184 (12.0)
	Maine	6/184 (3.3)
	New Hampshire	1/184 (0.5)
	Vermont	3/184 (1.6)
	Other	16/184 (8.7)
Clinician type	Nurse practitioner	44/184 (23.9)
	Primary care physician	40/184 (21.7)
	Infectious diseases physician	39/184 (21.1)
	Other specialist physician	23/184 (12.5)
	Resident or Fellow	22/184 (12.0)
	Physician assistant	6/184 (3.3)
	Other	10/184 (5.4)
Experience with HIV-infected patients	Percentage of patient panel with HIV-infection	15% (5–45%)
	Years caring for HIV-infected patients	10 (4–20)
	HIV-infected patients under direct care^a^	17 (2–80)
	HIV-infected patients cared for in typical month^a^	8 (1–30)
ART prescribing	HIV-infected patients initiated on ART in prior year	3 (3–15)
	ART initiation in prior year with main goal of reducing infectiousness^a^	1 (0–5)
	Percent of HIV-infected patients currently on ART^a^	90% (80–100%)

IQR, interquartile range; ART, antiretroviral treatment; LGBT, lesbian, gay, bisexual, and transgender.

^a^Restricted to ART-prescribing clinicians, i.e. practitioners who prescribe antiretroviral treatment to HIV-infected patients (n = 112).

Respondents reported that a median of 30% of their HIV-infected patients were female, 30% men who have sex with men (MSM), 15% persons who inject drugs, and 10% heterosexual men; 25% were Latino/a and 30% were Black. A median of 40% of their patients had Medicaid and 5% were uninsured. ART-prescribing clinicians had initiated a median of 3 patients on ART in the prior year. Nearly all HIV-infected patients were receiving ART (90%).

To assess non-response bias, survey completers were compared to all invited clinicians with respect to demographic and practice characteristics (S1 Table). Demographics and employment settings were similar between these 2 groups, though completers were more likely to be nurse practitioners (24% versus 15%) and have greater HIV care experience (median 10 years versus 6 years).

### Knowledge and Beliefs regarding Early ART

Among 105 ART-prescribing clinicians responding to the question about whether DHHS guidelines recommended ART for all HIV-infected patients, 64% participants correctly responded true, 25% responded false, and 11% were not sure. Nearly all ART-prescribing clinicians (95%) strongly agreed or agreed that early ART could slow the spread of HIV in a community and 88% indicated they considered a patient’s HIV transmission risk behaviors when recommending ART (Fig 1). However, 77% of respondents strongly agreed or agreed that they tended to defer ART if a patient was not ready to initiate, and some reported concerns that early ART could result in toxicities or resistant virus. One-fourth of clinicians perceived that patients with untreated depression or alcohol/substance abuse needed to have these illnesses under control before initiating ART. Participants indicated that they generally did not defer prescribing ART because of concerns about a patient’s ability to pay for ART, or because monitoring patients on ART required more staff, or because of limited financial support to pay for ART in their setting.

### Prescribing Intentions, Practices, and Experiences with Early ART

Over two-thirds (69%) of respondents would initiate ART in all patients irrespective of CD4+ count. Most clinicians would also initiate ART in patients who were receiving treatment for tuberculosis (82%), or who had hepatitis B (81%), hepatitis C (75%), HIV-associated nephropathy (89%), HIV-associated dementia (91%), or pregnancy (94%). When discussing initiation of ART with patients who have CD4+ count > 500 cells/μL, among the 108 ART-prescribing respondents, 68% always/often recommended initiation of ART to patients with CD4+ count > 500 cells/μL, but 17% reported these patients always/often objected to initiating, and 44% reported always/often trying to convince them to initiate early ART.

### Multivariable Modeling: Early ART

In a multivariable model adjusted for age, gender, and race/ethnicity, percent of patients who are HIV-infected was associated with intentions to prescribe early ART (aOR 1.16 per 10% increase in proportion of patients who are HIV infected, 95% CI 1.01–1.34), and infectious diseases specialty (versus primary care physician) was borderline associated with intentions to prescribe early ART (aOR 3.32, 95% CI 0.98–11.2) (Table 2). None of the other covariates entered into the model were associated with intentions to prescribe early ART. In additional models, none of these covariates were associated with awareness of current DHHS recommendations or having recommended ART always/often to patients with CD4+ count > 500 cells/μL.

**Table 2:** Factors associated with awareness, practices, and prescribing intentions regarding early antiretroviral treatment (ART) among ART-prescribing clinicians, New England, 2013.

		Model		
		Aware of DHHS Recommendations regarding Early ART (n = 102^a^)	More Likely to Recommend Early ART (n = 101)	Intend to Prescribe Early ART to All Patients (n = 103)
Practitioner Characteristics		aOR^b^ (95% CI)	aOR^c^ (95% CI)	aOR^b^ (95% CI)
Age (years)		0.96 (0.91 to 1.01)	0.98 (0.94 to 1.01)	0.98 (0.94 to 1.03)
Female gender		0.87 (0.33 to 2.30)	0.95 (0.44 to 2.06)	0.84 (0.33 to 2.16)
Clinician type	PCP	Ref	Ref	Ref
	ID Specialist	1.04 (0.30 to 3.59)	2.40 (0.89 to 6.43)	3.32 (0.98 to 11.2)
	ANP/PA	1.16 (0.29 to 4.68)	2.00 (0.64 to 6.19)	1.30 (0.36 to 4.70)
	All other	1.10 (0.24 to 5.13)	1.38 (0.39 to 4.84)	1.97 (0.48 to 8.02)
White/Caucasian vs. all other race/ethnicities		1.01 (0.30 to 3.39)	0.75 (0.30 to 1.92)	1.14 (0.35 to 3.65)
Percent of patients who are HIV-infected^d^		1.04 (0.90 to 1.20)	1.00 (0.89 to 1.11)	**1.16 (1.01 to 1.34)**

DHHS, Department of Health and Human Services; aOR, adjusted odds ratio; PCP, primary care physician; ID, infectious diseases; ANP, advanced nurse practitioner; PA, physician assistant. Significant results in bold.

^a^Three participants did not report race/ethnicity and were excluded from model.

^b^Multivariable logistic regression models adjusted for all other covariates from first 2 columns.

^c^Ordinal logistic regression model adjusted for all other covariates from first 2 columns. Ordered levels were never, rarely, sometimes, often, and always.

^d^Per 10% increase in percent of practice that is HIV-infected.

### PrEP Knowledge, Practices and Prescribing Intentions

Nearly all practitioners responding to questions about PrEP (n = 181) had heard of PrEP (89%). Seventy-one percent of participants identified a statement about FDA approval of PrEP [28] as correct, 27% were not sure, and 2% incorrectly responded that this was false. Three-fourths of respondents correctly identified that CDC guidance for PrEP had been issued, 24% were not sure, and 2% indicated that this was false. A minority of clinicians had prescribed PrEP (19%). The number of patients to whom clinicians had prescribed PrEP ranged from 0 to 80 patients. When asked if any providers had prescribed PrEP at their clinic/center, responses were yes (37%), no (26%), don’t know (32%), refuse to answer (1%), and not applicable (3%). Clinicians had prescribed PrEP to MSM (63%), HIV serodiscordant couples (63%), and persons who used post-exposure prophylaxis (37%), had a sexually transmitted infection (29%), changed sex partners frequently (29%), exchanged sex for money or drugs or other goods (29%), or used injection drugs (20%). Clinicians who had prescribed PrEP indicated their likelihood of prescribing PrEP in the future as very unlikely (14%), unlikely (11%), likely (43%), or very likely (31%). Practitioners who had not prescribed PrEP reported their likelihood of prescribing PrEP as very unlikely (13%), unlikely (30%), likely (45%), or very likely (13%).

### Beliefs about PrEP and Perceived Barriers to Prescribing in Clinical Settings

**Fig 2:**
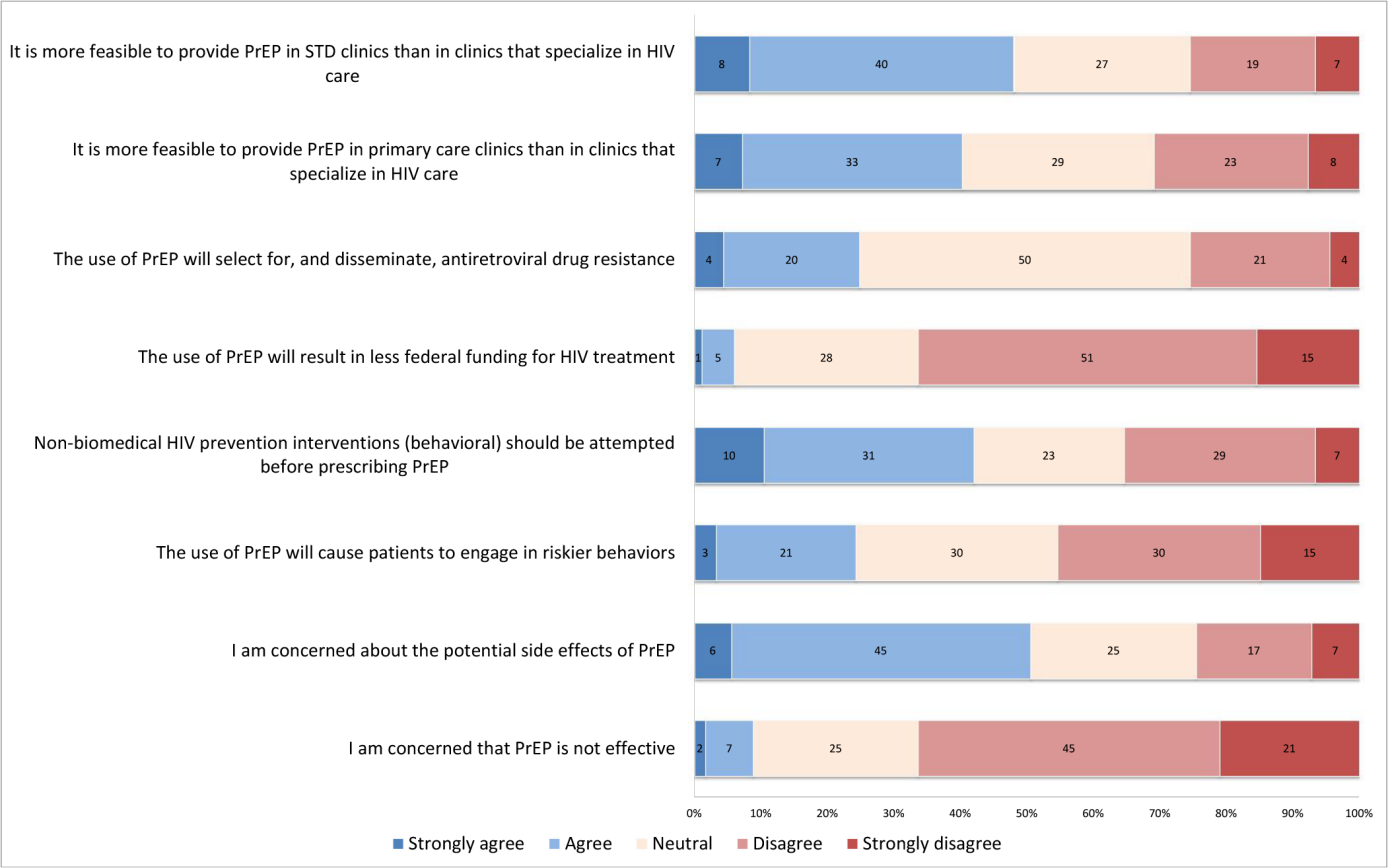
Clinician beliefs regarding pre-exposure prophylaxis (n = 181), New England, 2013. Participants indicated their degree of agreement (strongly agree, agree, neutral, disagree, strongly disagree) to statements about pre-exposure prophylaxis. Numbers within bars represent the percentage of respondents selecting each response category. Blue shading represents agreement, neutral shading represents neutrality, and red shading represents disagreement.

Almost all of the respondents believed that PrEP was effective (only 6% strongly agreed or agreed that PrEP was not effective; Fig 2). However, substantial proportions of clinicians were concerned that PrEP use could potentially be associated with side effects, drug resistance, or increased risk behaviors. One-third of practitioners thought that behavioral interventions should be utilized before PrEP. Some participants believed PrEP provision would be more feasible in primary care settings or STD clinics than clinics specializing in HIV care.

**Fig 3:**
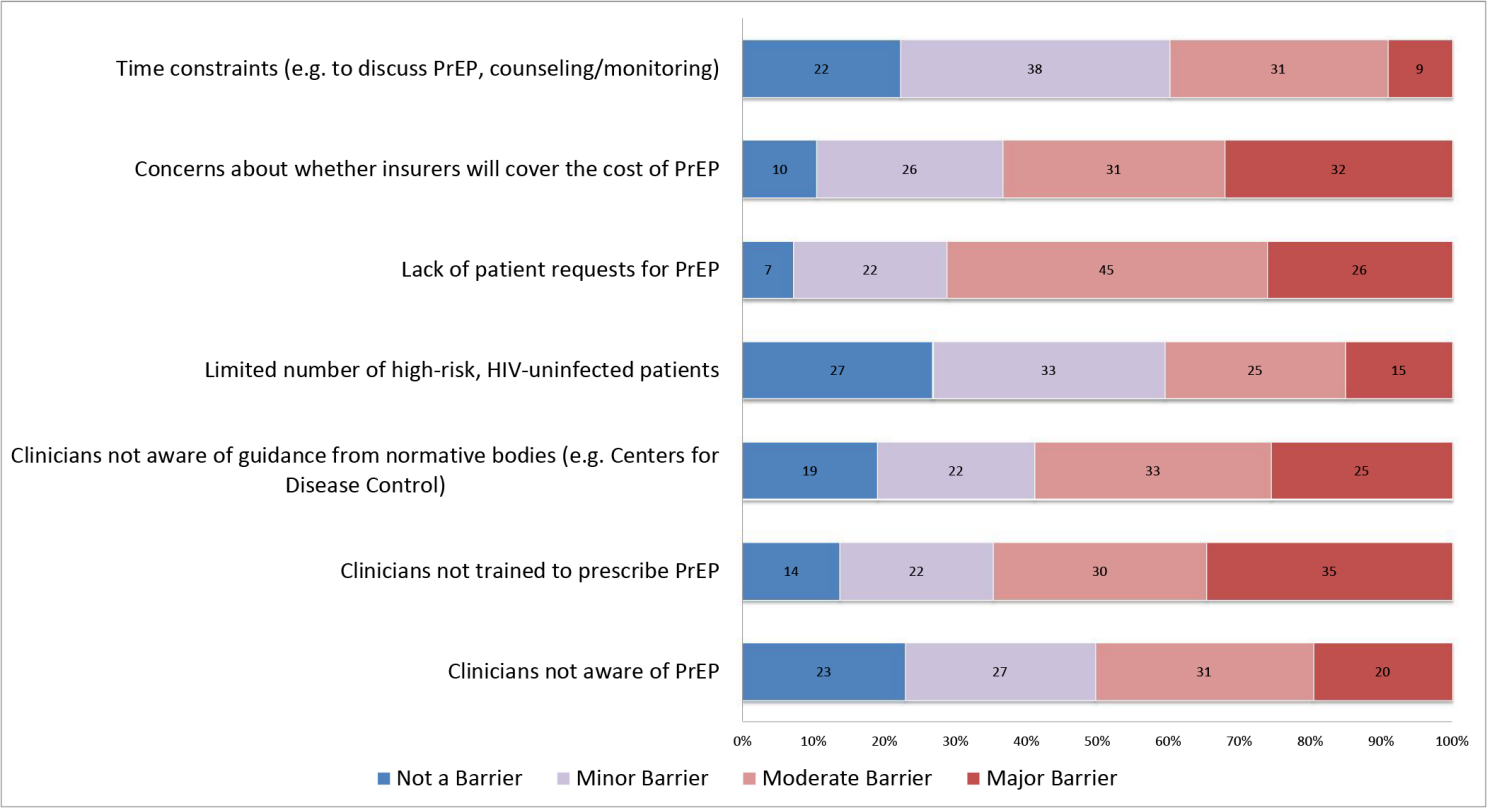
Clinicians’ perceived barriers to prescribing pre-exposure prophylaxis (n = 155), New England, 2013. Numbers within bars represent the percentage of participants selecting each response category. Data are restricted to clinicians from specialties for which PrEP prescribing may be feasible, i.e., those that involve provision of longitudinal medical care.

Participants perceived numerous practical barriers to prescribing PrEP, including lack of patient requests for PrEP, concerns about insurance coverage, and limited practitioner training in PrEP provision, among other barriers (Fig 3).

### Multivariable Modeling: PrEP

Older age was associated with awareness of CDC guidance regarding PrEP (aOR 1.06, 95% CI 1.01 to 1.11) and being likely to prescribe PrEP in the future (adjusted proportional OR 1.03, 95% CI 1.00–1.07) (Table 3). Older age was also associated with having prescribed PrEP (aOR 1.05, 95% CI 1.00 to 1.09), whereas female gender (aOR 0.26, 95% CI 0.10–0.71) and white race (aOR 0.32, 95% CI 0.10–1.06) were associated with having not prescribed PrEP. Individuals with a higher percentage of HIV-infected patients indicated they were more likely to prescribe PrEP (aOR 1.19 per 10% increase in proportion of patients who are HIV infected, 95% CI 1.06–1.34).

**Table 3:** Factors associated with awareness, experience, and prescribing intentions regarding pre-exposure prophylaxis (PrEP) among healthcare providers, New England, 2013.

		Model		
		Aware of CDC Recommendations(n = 146)	Have Prescribed PrEP (n = 145^a^)	More Likely to Prescribe PrEP in Future(n = 118^a^ ^,^ ^b^)
Clinician Characteristics		aOR^c^(95% CI)	aOR^c^ (95% CI)	aOR^d^ (95% CI)
Age (years)		**1.06 (1.01 to 1.11)**	**1.05 (1.00 to 1.09)**	**1.03 (1.00 to 1.07)**
Female		1.33 (0.47 to 3.80)	**0.26 (0.10 to 0.71)**	0.71 (0.31 to 1.61)
Clinician type	PCP	Ref	Ref	Ref
	ID Specialist	6.21 (0.67 to 57.3)	0.82 (0.20 to 3.29)	0.64 (0.24 to 1.68)
	ANP/PA	0.34 (0.10 to 1.21)	2.59 (0.65 to 10.2)	0.78 (0.28 to 2.19)
	All other	1.18 (0.30 to 4.62)	3.22 (0.74 to 14.0)	1.95 (0.58 to 6.60)
White/Caucasian vs. all other race/ethnicities		1.07 (0.31 to 3.76)	0.32 (0.10 to 1.06)	0.68 (0.24 to 1.96)
Percent of patients who are HIV-infected^e^		1.18 (0.97 to 1.44)	1.02 (0.88 to 1.18)	**1.19 (1.06 to 1.34)**

CDC, Centers for Disease Control and Prevention; aOR, adjusted odds ratio; PCP, primary care physician; ID, infectious diseases; NP, advanced nurse practitioner; PA, physician assistant. Significant results in bold.

^a^Analyses restricted to providers from specialties that involve longitudinal care of medical patients.

^b^Among clinicians who had not previously prescribed PrEP.

^c^Multivariable logistic regression models adjusted for all other covariates from first column.

^d^Ordinal logistic regression model adjusted for all other covariates from first 2 columns. Ordered levels were very unlikely, unlikely, likely, and very likely.

^e^Per 10% increase in percent of practice that is HIV-infected.

## Discussion

This study suggests that a majority of HIV clinicians have adopted prescribing practices consistent with implementing HIV Treatment as Prevention. In contrast, few clinicians have integrated PrEP provision into practice despite preliminary CDC guidance at the time of the survey [29–31]. When interpreted through a conceptual framework of how medical innovations disseminate in healthcare, the diffusion of innovation theory [32], these results suggest that Treatment as Prevention may have reached a tipping point, such that adoption of this approach is likely to occur among all but the most conservative clinicians [33]. However, as dissemination of PrEP is at a stage when only a small number of innovative practitioners (“early adopters”) have prescribed PrEP, widespread adoption of this intervention is possible but not yet certain [33].

Nearly all practitioners believed that early ART and PrEP were effective at reducing HIV transmission. However, substantial numbers of clinicians were concerned about potential negative consequences with these strategies, such as adverse medication effects and dissemination of viral drug resistance, as has been found in prior surveys of clinicians [12–14, 17, 19]. Informing practitioners that HPTN 052 and observational studies suggest that earlier initiation of ART is associated with improved clinical outcomes [4, 5], and that numerous studies have demonstrated that PrEP use is generally safe and rarely associated with drug resistance [7–10, 34, 35], could reassure clinicians that these strategies are likely to benefit patients despite theoretical harms.

Clinicians also perceived practical barriers to prescribing early ART and PrEP. For early ART, these included patients’ psychosocial comorbidities and incomplete readiness for ART. For PrEP, practitioners perceived limited patient interest and felt that they needed more clinical training. As nearly one-third of clinicians did not intend to prescribe early ART, and 40% of clinicians who had not prescribed PrEP did not envision doing so in the future, developing pragmatic solutions for overcoming perceived barriers should be prioritized.

The finding that 69% of clinicians intended to prescribe early ART suggests a marked evolution in provider practices over the past few years, as only 14% of HIV clinicians in 2 mid-Atlantic cities reported similar intentions as recently as 2011 [12]. Clinicians may have adopted early ART into practice in response to updated guidelines recommending early ART [6] or to data supporting the individual health [4, 5, 36] or transmission-related benefits [2, 37] of this approach. In San Francisco, ART has been offered to all HIV-infected patients receiving care through public health clinics since 2010. However, patients in San Francisco with CD4+ counts > 500 cells/μL had already tended to initiate early ART prior to 2010, which suggests that clinicians had shifted towards earlier initiation of ART prior to official changes in policy [38]. Further studies to understand the types of data or guidance that most effectively influence clinician prescribing decisions, particularly among practitioners who may be cautious about early ART (i.e., late adopters, or “traditionalists” [32, 33]), could accelerate the implementation of Treatment as Prevention.

One-fourth of clinicians were unfamiliar with CDC guidance regarding PrEP provision more than 2 years after publication [11]. As practitioners who affiliate with an AIDS Education and Training Center would be expected to have greater awareness of novel HIV-related guidance than a general population of clinicians, this result highlights an ongoing need to disseminate information about PrEP. Only one-fifth of clinicians had prescribed PrEP and most cited practical barriers to prescribing, which is consistent with prior studies of healthcare practitioners [13, 15, 17, 20]. Perceived barriers included their concern that they needed more training; they also expressed uncertainties about insurance coverage, time constraints, and a perception that few patients in their care were at high-risk for HIV acquisition. The nature of these concerns suggests busy practitioners may benefit from trainings that summarize guidelines, provide strategies for overcoming any insurance-related barriers to prescribing PrEP, and offer simple tools for identifying appropriate candidates for PrEP. As knowledge transfer among professional peers is important for the diffusion of medical innovations [39], enhanced visibility of early adopters who have successfully prescribed PrEP could also engage clinicians who are more cautious [33]. As this study found that lack of patient requests was also a barrier to prescribing PrEP, campaigns that empower patients to discuss PrEP with their clinicians could complement efforts to optimize provider practices.

Clinicians with increasing percentages of HIV-infected patients in their panels reported greater intentions to prescribe early ART and PrEP, but they did not indicate a greater likelihood of having prescribed these interventions. Therefore, clinicians who are more experienced with HIV care may need resources to help them translate their positive intentions into prescribing practices. For clinicians who are less familiar with HIV care, interventions may need to focus on fostering positive prescribing intentions as a first-step towards altering their prescribing behaviors.

Infectious diseases specialists were more likely to recommend early ART and report intentions to prescribe early ART than generalist physicians. As one-third of experienced HIV practitioners in the US are nearing retirement, professional societies for HIV practitioners have advocated for expanding the role of generalists in HIV care [40]. Therefore, efforts to optimize ART-prescribing practices within generalist networks may become increasingly important. Interestingly, infectious diseases specialists were more likely than other clinicians to be aware of CDC guidance for PrEP, but ANP/PAs and “Other” clinicians were more likely than primary care providers to have prescribed PrEP. While definitive conclusions are difficult due to the small sample size and wide confidence intervals, these results seem to suggest a need to learn more about the perspectives and experiences of ANPs within the NEAETC network. Older clinicians were also more likely to have prescribed PrEP and to have indicated future prescribing intentions, so efforts to engage younger clinicians may also be needed. A more comprehensive understanding of how clinician demographics affect prescribing patterns for PrEP will be important, given that the reasons why white and female clinicians reported less PrEP experience were not evident.

The design of this study has limitations. A major potential limitation is that convenience sampling and a low response rate could introduce non-response bias; representative surveys would limit the potential for this bias. However, non-response bias is less likely given similar demographic and practice characteristics among respondents and non-respondents [41, 42]. The small sample size affects the precision of odds ratios for modeling analyses, so care should be taken when interpreting them, including not to dismiss large associations that are not significant, and not to ignore wide confidence intervals for statistically significant associations. As a result of the small sample size, some clinician categories had to be combined (potentially masking some associations), other clinician characteristics had to be omitted from the analysis, and a more detailed exploration of interactions among covariates (e.g., for mutually exclusive categories of profession and HIV experience) could not be considered. As participants were from New England and affiliated with an AIDS Education Training Center, and respondents had variable degrees of specialization with HIV care, study findings may not be generalizable to other clinicians, such as those clinicians who provide care almost exclusively to HIV-infected patients. Self-reported clinical practices may not accurately reflect prescribing behaviors, given potential confounders like social desirability bias, so independent confirmation of practices would strengthen confidence in the study findings. Clinician norms may have evolved since the end of enrollment for this study in late 2013, as CDC released formal PrEP guidelines in May 2014, so ongoing assessments of practitioner experiences are needed.

## Conclusion

This study suggests that most clinicians, though not all, have undergone a paradigm shift towards earlier initiation of ART over the past several years, but that adoption of PrEP provision into clinical practice has been more gradual. Interventions to address clinicians’ theoretical and practical concerns regarding early ART and PrEP, as well as campaigns that enhance patient readiness for ART and empower patients to discuss PrEP with their clinicians, could optimize implementation of Treatment as Prevention and PrEP.

## Supporting Information

S1 Table Characteristics of survey completers (n = 184) versus all invited practitioners (n = 1637), New England, 2013. (DOCX)

S1 Appendix Study dataset. (XLS)
