# Supplementary material for: Knowledge, Beliefs and Practices Regarding Antiretroviral Medications for HIV Prevention: Results from a Survey of Healthcare Providers in New England
Source: PLoS One. 2015 Jul 6;10(7):e0132398. doi: 10.1371/journal.pone.0132398 (PMC4492498; doi:10.1371/journal.pone.0132398)
Supplement: S1 Table — (DOCX) [file pone.0132398.s001.docx]

**S1 Table.** Characteristics of survey completers (n=184) versus all invited practitioners (n=1637), New England, 2013.

| **Characteristics** | | **Survey Completers** | **All Invited Practitioners** | ***P*-value**^a^ |
| --- | --- | --- | --- | --- |
|  |  | n^b^ (%) | n^b^ (%) |  |
| Female |  | 103/181 (56.9%) | 870/1637 (53.2%) | 0.35 |
| Race^c^ | White | 142/174 (81.6%) | 1265/1641 (77.1%) | 0.21 |
|  | Asian | 21/174 (12.1%) | 237/1641 (14.4%) |  |
|  | Black or African American | 9/174 (5.2%) | 76/1641 (4.6%) |  |
|  | Other | 2/174 (1.2%) | 63/1641 (3.8%) |  |
| Hispanic or Latino/a |  | 6/178 (3.4%) | 78/1,637 (4.8%) | 0.57 |
| Employment setting | Suburban or urban | 172/182 (94.5%) | 1462/1595 (91.7%) | 0.25 |
|  | Rural | 10/182 (5.5%) | 133/1595 (8.3%) |  |
| Provider type^d^ | Physician | 126/184 (68.5%) | 1339/1637 (81.8%) | 0.003 |
|  | Nurse Practitioner | 44/184 (23.9%) | 242/1637 (14.8%) |  |
|  | Physician Assistant | 6/184 (3.3%) | 56/1637 (3.4%) |  |
| Years caring for HIV-infected patients, median (IQR) |  | 10 (4-20) | 6 (1-17) | <0.001 |

IQR, interquartile range.

^a^Fisher’s exact test for categorical variables and Wilcoxon rank sum test for continuous variables.

^b^Number responding affirmatively to each question/total number of respondents for each question, given missing data.

^c^For Race, denominator is 1641 because participants could select more than 1 category.

^d^Percentages do not total to 100%, as 8 survey completers identified as “Other.”
